# Supplementary material for: Age-Dependence and Aging-Dependence: Neuronal Loss and Lifespan in a C. elegans Model of Parkinson’s Disease
Source: Biology (Basel). 2017 Dec 23;7(1):1. doi: 10.3390/biology7010001 (PMC5872027; doi:10.3390/biology7010001)
Supplement: Supplementary file 1 [file biology-07-00001-s001.zip › supplement_.pdf]

Supplementary Information

Age-dependence and aging-dependence: Neuronal loss and lifespan in a *C. elegans* model of Parkinson's disease

Javier Apfeld [1,*] and Walter Fontana [2,*]

[1] Biology Department, Northeastern University, Boston MA 02115, USA; j.apfeld@northeastern.edu
[2] Department of Systems Biology, Harvard Medical School, Boston MA 02115, USA; walter@hms.harvard.edu
* Correspondence: j.apfeld@northeastern.edu and walter@hms.harvard.edu

Figure S1: Independence of neuronal loss and organismic death in wild type animals.

(**a-d**) No difference in lifespan was observed when 3,397 seven-day old wild-type animals expressing α-synuclein in the dopaminergic neurons were grouped according to whether each of their cephalic neurons was present (blue line) or absent (red line). For statistics see Table S2. (**e-h**) Using computer simulations (Figure 3), we predicted how the mean lifespans of seven-day old animals with present (blue line) and absent (red line) cephalic neurons are influenced by the Spearman's rank correlation between the timing of neuronal loss and lifespan. The shaded areas represent the 95% point-wise confidence interval of the predicted mean lifespans. The observed mean lifespans (dotted lines) are only consistent with correlations values near zero. (**i-m**) The statistical power of our experiment is the fraction of simulations with a statistically significant ($P < 0.05$) difference in survival between the neuron present and absent groups. The shaded region denotes the Spearman's rank correlation values where statistical power is greater than 95% (dotted line). Panel m shows the aggregate power, the probability that no difference in lifespan between present and absent groups would be observed for any of the four neurons. For completeness, we are re-presenting the wildtype data in Figure 2 for CEPDL and CEPVL.

Figure S2: Independence of neuronal loss and organismic death in wild type animals.

In wild-type animals expressing α-synuclein in the dopaminergic neurons, those that exhibit any neurodegeneration at day 7 of adulthood ("< Four" cephalic neurons) have lifespans indistinguishable from those that exhibit no neurodegeneration ("= Four" cephalic). Additionally, no difference in lifespan is observed when these animals are grouped according to whether each of the four cephalic neurons is present or absent (Figures 2 and S1a-d), or based on the degree of neurodegeneration within a group of cephalic neurons. The cephalic cell group (CEP) refers to all four cephalic neurons. Dorsal (CEPD) and ventral (CEPV) pairs consist of the corresponding left and right neurons. For statistics see Table S2.

Figure S3: Independence of neuronal loss and organismic death in *age-1* mutants.

(**a-d**) No difference in lifespan was observed when 542 seven-day old *age-1(hx546)* mutants expressing α-synuclein in the dopaminergic neurons were grouped according to whether each of their cephalic neurons was present (blue line) or absent (red line). For statistics see Table S2. (**e-h**) Using computer simulations (Figure 3), we predicted how the mean lifespans of seven-day old animals with present (blue line) and absent (red line) cephalic neurons are influenced by the Spearman's rank correlation between the timing of neuronal loss and lifespan. The shaded areas represent the 95% point-wise confidence interval of the predicted mean lifespans. The observed mean lifespans (dotted lines) are only consistent with correlations of low magnitude. (**i-m**) The statistical power of our experiment is the fraction of simulations with a statistically significant ($P < 0.05$) difference in survival between the neuron present and absent groups. The shaded region denotes the Spearman's rank correlation values where statistical power is greater than 95% (dotted line). Panel m shows the aggregate power, the probability that no difference in lifespan between present and absent groups would be observed for any of the four neurons.For completeness, we are re-presenting the *age-1* data in Figure 5 for CEPDL and CEPVL.

Figure S4: Independence of neuronal loss and organismic death in *age-1* mutants.

In *age-1(hx546)* mutants expressing α-synuclein, those that exhibit any neurodegeneration at day 7 of adulthood ("< Four" cephalic neurons) have lifespans indistinguishable from those that exhibit no neurodegeneration ("= Four" cephalic). Additionally, no difference in lifespan is observed when these animals are grouped according to whether each of the four cephalic neurons is present or absent (Figures 5 and S3a-d), or based on the degree of neurodegeneration within a group of cephalic neurons. The cephalic cell group (CEP) refers to all four cephalic neurons. Dorsal (CEPD) and ventral (CEPV) pairs consist of the corresponding left and right neurons. For statistics see Table S2.

Video S1: Simulations of the joint variation of the timing of neuronal loss and lifespan.

We performed computer simulations to investigate how the correlation structure of the timing of neuronal loss ("neuronspan") and lifespan would affect the survival curves of seven-day old animals with present or absent cephalic neurons. The video shows a single simulation for each value of the Spearman's rank correlation using the data for CEPVL in wild-type animals (Figure 2b,d,f). (**left**) We represented the correlation structure of lifespan and CEPVL neuronspan using randomly generated copulas with specific Spearman's rank correlations. We partitioned the simulated observations into "cell present" (2,881 blue dots) and "cell absent" (516 red dots) groups, assigning the lowest neuronspan ranks to the latter. Each simulated observation was assigned a lifespan based on the rank order of the 3,397 observed lifespans. (**right**) Survival curves of the "cell present" (blue) and "cell absent" groups generated using the procedure described in the left panel. This procedure allowed us to calculate confidence intervals for the predicted mean lifespans of each group (Figure 2d), and determine the likelihood that the two groups exhibit a statistically significant difference in survival (Figure 2f), as a function of the Spearman's rank correlation between neuronspan and lifespan.

Table S1: Age-dependent neurodegeneration in wild-type animals expressing α-synuclein in dopaminergic neurons.

Statistical analysis for Figure 1.

Table S2: Independence of lifespan and cephalic neurodegeneration in animals expressing α-synuclein in dopaminergic neurons.

Statistical analysis for Figures 2 and 5.

Table S3: Statistical analysis of adult lifespans of animals expressing α-synuclein in dopaminergic neurons.

Statistical analysis for Figure 4b.

Table S4: Percent of cephalic neurons missing in wild type and mutants expressing α-synuclein in dopaminergic neurons.

Statistical analysis for Figure 4a.

Table S5: Percent of cephalic neurons missing in wild type and *daf-2(e1370)* mutants expressing α-synuclein in dopaminergic neurons.

Figure S1

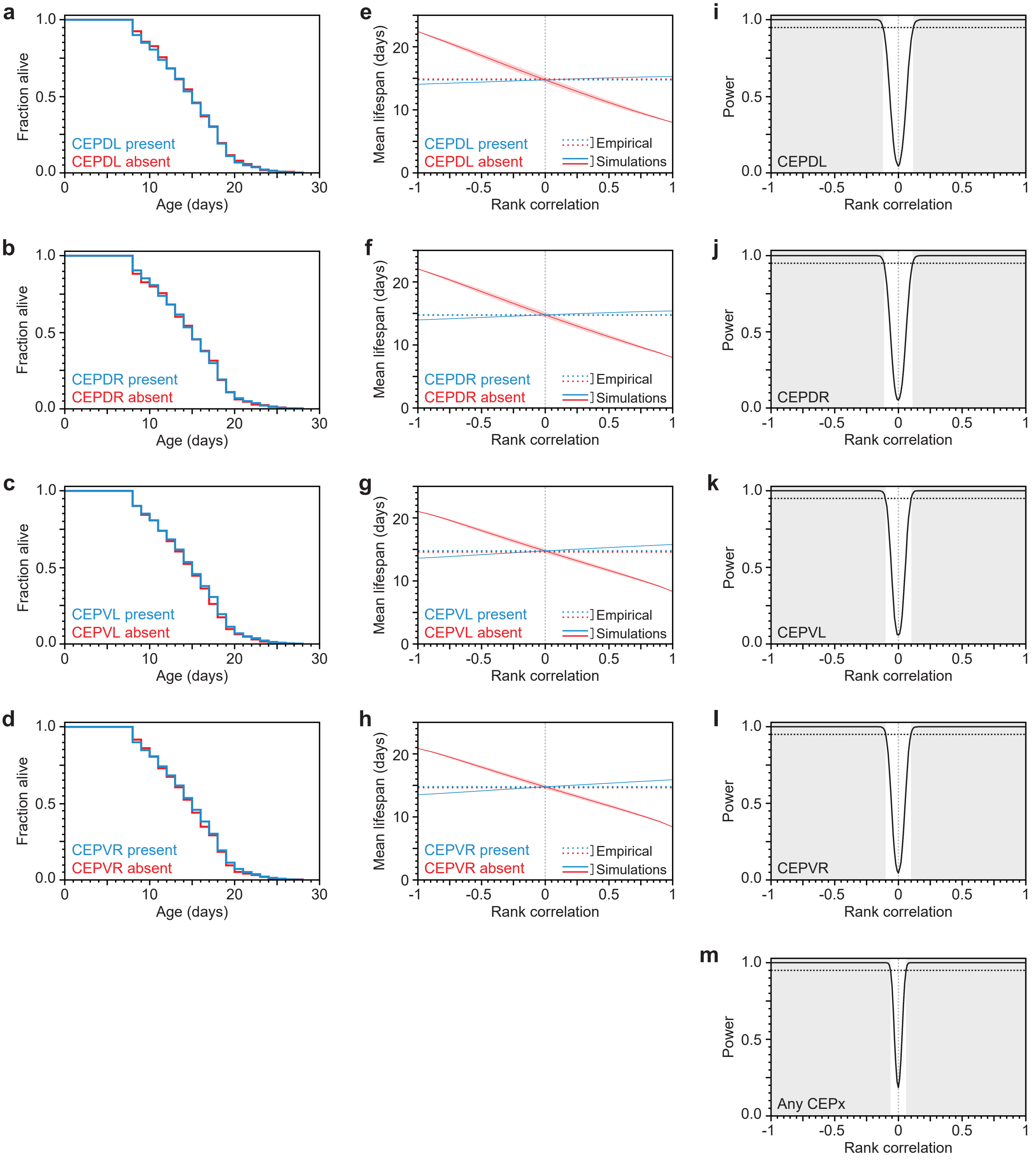

a
b
c
d
e
f
g
h
i
j
k
l
m
Fraction alive
Age (days)
CEPDL present
CEPDL absent
CEPDR present
CEPDR absent
CEPVL present
CEPVL absent
CEPVR present
CEPVR absent
Mean lifespan (days)
Rank correlation
Empirical
Simulations
Power
CEPDL
CEPDR
CEPVL
CEPVR
Any CEPx

Figure S2

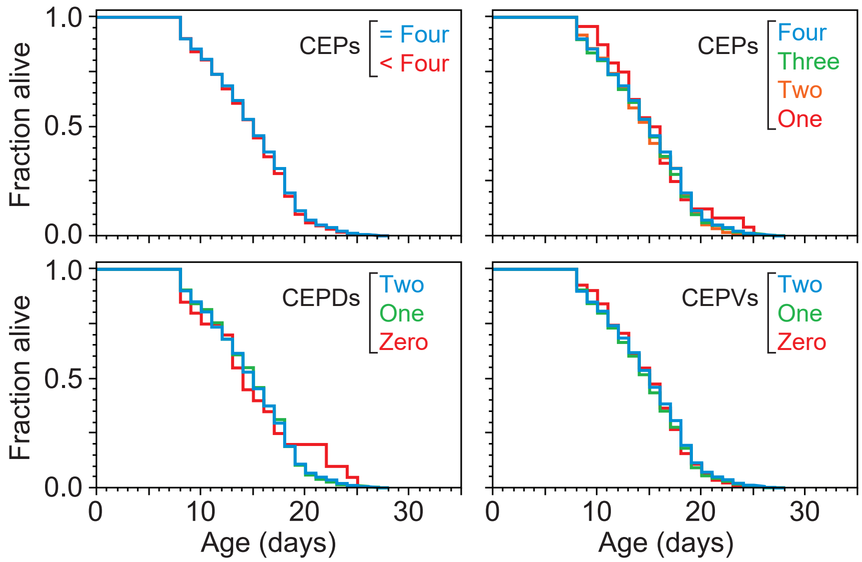
Fraction alive
1.0
0.5
0.0
CEPs
= Four
< Four
CEPs
Four
Three
Two
One
CEPDs
Two
One
Zero
CEPVs
Two
One
Zero
0
10
20
30
Age (days)

Figure S3

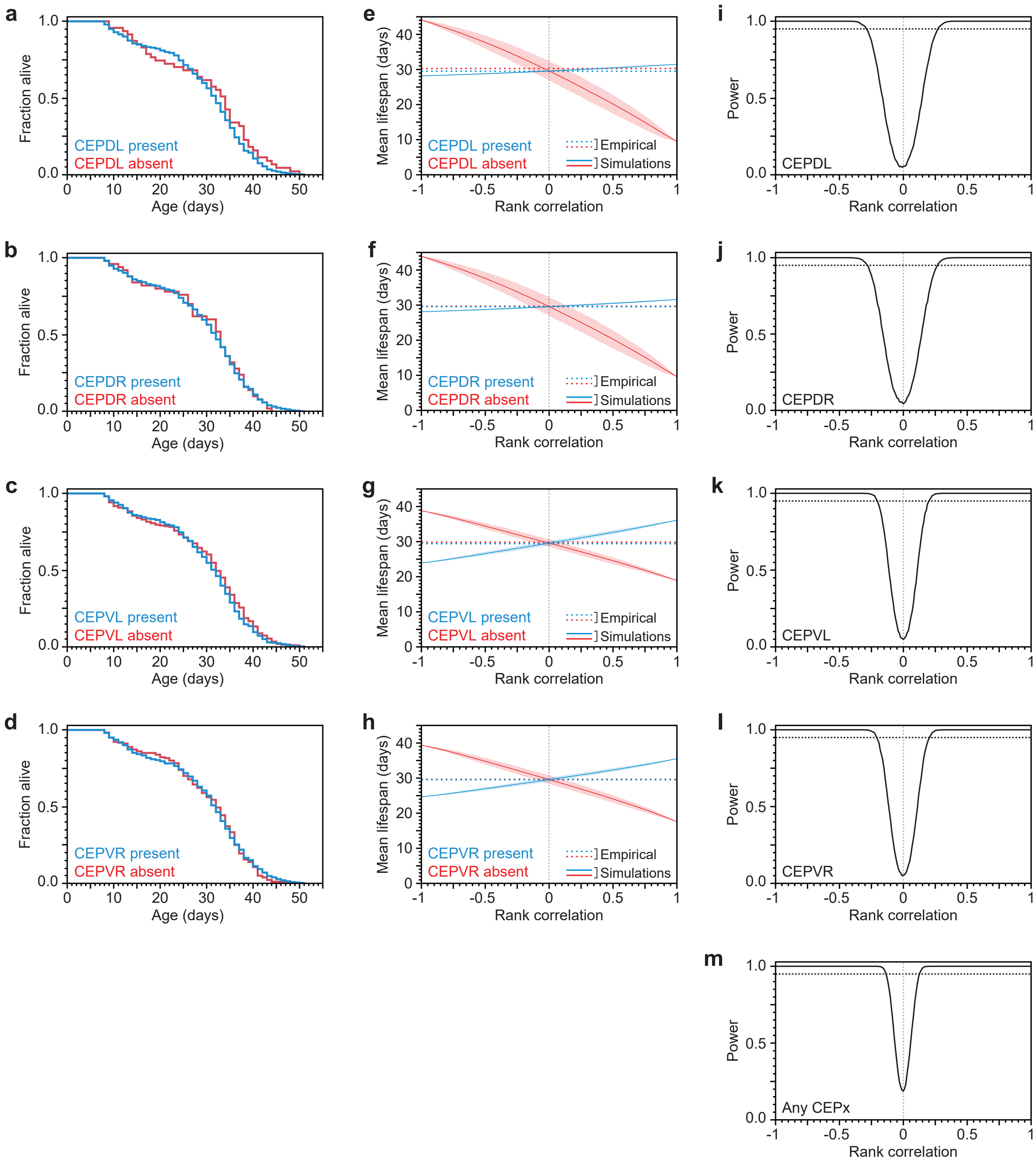
a
Fraction alive
Age (days)
CEPDL present
CEPDL absent
b
CEPDR present
CEPDR absent
c
CEPVL present
CEPVL absent
d
CEPVR present
CEPVR absent
e
f
g
h
Mean lifespan (days)
Rank correlation
Empirical
Simulations
i
j
k
l
m
Power
CEPDL
CEPDR
CEPVL
CEPVR
Any CEPx

Figure S4

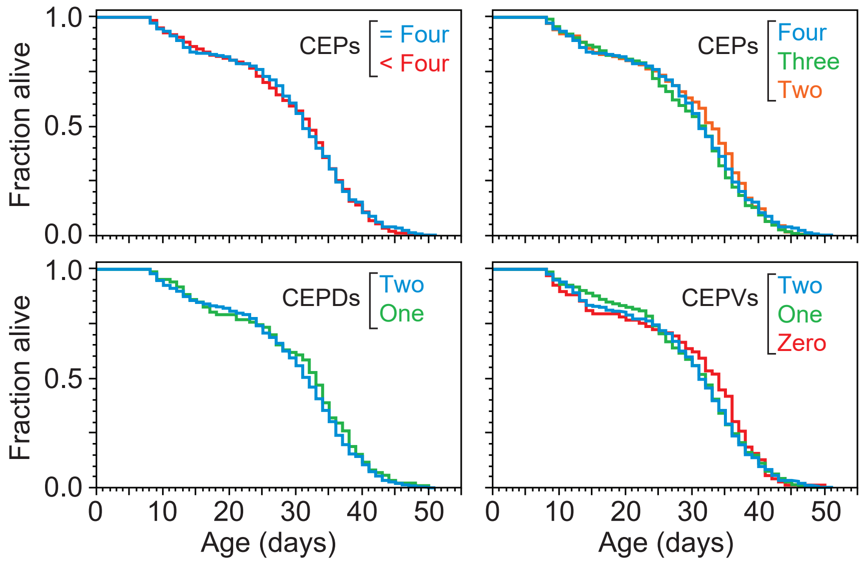

CEPs
= Four
< Four
CEPs
Four
Three
Two
CEPDs
Two
One
CEPVs
Two
One
Zero
Fraction alive
Age (days)

Table S1: Age-dependent neurodegeneration in wild-type animals expressing α-synuclein in dopaminergic neurons.

		Control group			α-synuclein group			
Cell	Age cell observed	Cells missing (%)	n	Comparisons across age (Chi-square test)	Cells missing (%)	n	Comparisons across age (Chi-square test)	Comparisons across group (Fisher's Exact test)
CEPDL	L4	0.0 %	(101)	*P* > 0.05	0.0 %	(110)	*P* < 0.0001	*P* > 0.05
	Day 0	0.0 %	(110)		0.0 %	(200)		*P* > 0.05
	Day 1	0.0 %	(100)		0.5 %	(223)		*P* > 0.05
	Day 4	0.0 %	(122)		1.9 %	(206)		*P* > 0.05
	Day 7	0.0 %	(115)		9.2 %	(217)		*P* = 0.0004
CEPDR	L4	0.0 %	(101)	*P* > 0.05	0.0 %	(110)	*P* < 0.0001	*P* > 0.05
	Day 0	0.0 %	(110)		0.0 %	(200)		*P* > 0.05
	Day 1	0.0 %	(100)		0.5 %	(223)		*P* > 0.05
	Day 4	0.0 %	(122)		1.5 %	(206)		*P* > 0.05
	Day 7	0.0 %	(115)		7.4 %	(217)		*P* = 0.0036
CEPVL	L4	0.0 %	(101)	*P* > 0.05	0.0 %	(110)	*P* < 0.0001	*P* > 0.05
	Day 0	0.0 %	(110)		0.0 %	(200)		*P* > 0.05
	Day 1	0.0 %	(100)		1.4 %	(223)		*P* > 0.05
	Day 4	0.0 %	(122)		4.9 %	(206)		*P* = 0.034
	Day 7	0.0 %	(115)		17.1 %	(217)		*P* < 0.0001
CEPVR	L4	0.0 %	(101)	*P* > 0.05	0.0 %	(110)	*P* < 0.0001	*P* > 0.05
	Day 0	0.0 %	(110)		0.0 %	(200)		*P* > 0.05
	Day 1	0.0 %	(100)		0.9 %	(223)		*P* > 0.05
	Day 4	0.0 %	(122)		4.4 %	(206)		*P* > 0.05
	Day 7	0.0 %	(115)		19.4 %	(217)		*P* < 0.0001

Table S2: Independence of lifespan and cephalic neurodegeneration in animals expressing α-synuclein in dopaminergic neurons.

α-synuclein	Genotype	Age cell observed	Class of grouping variable	Grouping variable	Grouping variable value	Mean ± s.e.m. (days)	75th percentile [a] (days)	Number of animals that died/total [b]	Survival functions equal? (Wilcoxon test)
Yes	wild type	Day 7	Individual cell	CEPDL	Cell present	14.8 ± 0.1	18	3064/3116	*P* > 0.05
					Cell absent	14.9 ± 0.3	18	277/281	
				CEPDR	Cell present	14.8 ± 0.1	18	3016/3066	*P* > 0.05
					Cell absent	14.7 ± 0.2	18	325/331	
				CEPVL	Cell present	14.8 ± 0.1	18	2835/2881	*P* > 0.05
					Cell absent	14.6 ± 0.2	18	506/516	
				CEPVR	Cell present	14.8 ± 0.1	18	2785/2833	*P* > 0.05
					Cell absent	14.6 ± 0.2	18	556/564	
			Cell pair	CEPD	Both cells present	14.8 ± 0.1	18	2759/2805	*P* > 0.05
					One cell present	14.8 ± 0.2	18	562/572	
					No cell present	14.9 ± 1.2	18	20/20	
				CEPV	Both cells present	14.8 ± 0.1	18	2361/2400	*P* > 0.05
					One cell present	14.6 ± 0.1	18	898/914	
					No cell present	14.8 ± 0.1	18	82/83	
			Cell group	CEP	Four cells present	14.8 ± 0.1	18	1954/1987	*P* > 0.05
					Three cells present	14.7 ± 0.1	18	1134/1152	
					Two cells present	14.6 ± 0.3	18	229/234	
					One cell present	15.3 ± 0.9	18	24/24	
					Four cells present	14.8 ± 0.1	18	1954/1987	*P* > 0.05
					Fewer than four cells present	14.7 ± 0.1	18	1387/1410	
	age-1(hx546)	Day 7	Individual cell	CEPDL	Cell present	29.5 ± 0.5	36	494/495	*P* > 0.05
					Cell absent	30.3 ± 1.6	38	46/47	
				CEPDR	Cell present	29.6 ± 0.5	36	490/492	*P* > 0.05
					Cell absent	29.7 ± 1.4	37	50/50	
				CEPVL	Cell present	29.4 ± 0.5	36	335/336	*P* > 0.05
					Cell absent	29.9 ± 0.7	38	205/206	
				CEPVR	Cell present	29.5 ± 0.5	37	360/362	*P* > 0.05
					Cell absent	29.7 ± 0.7	37	180/180	
			Cell pair	CEPD	Both cells present	29.5 ± 0.5	36	449/450	*P* > 0.05
					One cell present	30.0 ± 1.1	38	86/87	
					No cell present	29.4 ± 5.6	38	5/5	
				CEPV	Both cells present	29.3 ± 0.7	36	224/225	*P* > 0.05
					One cell present	29.7 ± 0.6	36	247/248	
					No cell present	29.8 ± 1.3	38	69/69	
			Cell group	CEP	Four cells present	29.6 ± 0.7	36	188/189	*P* > 0.05
					Three cells present	29.2 ± 0.6	36	236/236	
					Two cells present	30.1 ± 1.0	37	105/106	
					One cell present	34.3 ± 4.3	41	9/9	
					Zero cells present	26 ± 12	38	2/2	
					Four cells present	29.6 ± 0.7	36	188/189	*P* > 0.05
					Fewer than four cells present	29.9 ± 0.5	37	352/353	

a The 75th percentile is the age when the fraction of animals alive reaches 0.25.

b The total number of observations equals the number of animals that died plus the number censored. Animals that crawled off the plate or ruptured were censored at the time of the event. This step incorporates those animals until the censor date, and is necessary to avoid the loss of information.

Table S3: Statistical analysis of adult lifespans of animals expressing α-synuclein in dopaminergic neurons.

α-synuclein	Genotype	Mean ± s.e.m. (days)	75th percentile [a] (days)	Number of animals that died/total [b]	Survival function equal to wild type? (Wilcoxon test)	Survival function equal to *age-1(hx546)*? (Wilcoxon test)	Survival function equal to *daf-16(mu86); age-1(hx546)*? (Wilcoxon test)
Yes	wild type	10.5 ± 0.4	14	92/93			
	age-1(hx546)	27.7 ± 1.0	36	109/110	$P < 0.0001$		
	daf-16(mu86); age-1(hx546)	10.8 ± 0.4	13	97/100		$P < 0.0001$	
	daf-16(mu86); age-1(hx546); Pintestine::daf-16(+)	14.9 ± 0.6	20	104/108			$P < 0.0001$

a The 75th percentile is the age when the fraction of animals alive reaches 0.25.

b The total number of observations equals the number of animals that died plus the number censored. Animals that crawled off the plate or ruptured were censored at the time of the event. This step incorporated those animals until the censor date, and was necessary to avoid the loss of information.

Table S4: Percent of cephalic neurons missing in wild type and mutants expressing α-synuclein in dopaminergic neurons.

			Control group			α-synuclein group						
Genotype	Cell	Age	Cells missing (%)	n	Comparisons across age (Fisher's Exact test)	Cells missing (%)	n	Comparisons across age (Fisher's Exact test)	Comparisons to wild type (Fisher's Exact test)	Comparisons to *age-1(hx546)* (Fisher's Exact test)	Comparisons to *daf-16(mu86); age-1(hx546)* (Fisher's Exact test)	Comparisons across group (Fisher's Exact test)
wild type	CEPDL	Day 0	0.0 %	(136)	*P* > 0.05	0.0 %	(136)	*P* = 0.004				*P* > 0.05
		Day 7	0.0 %	(226)		6.6 %	(320)					*P* = 0.0001
	CEPDR	Day 0	0.0 %	(136)	*P* > 0.05	0.0 %	(136)	*P* = 0.004				*P* > 0.05
		Day 7	0.0 %	(226)		6.3 %	(320)					*P* = 0.0001
	CEPVL	Day 0	0.0 %	(136)	*P* > 0.05	0.0 %	(136)	*P* < 0.0001				*P* > 0.05
		Day 7	0.0 %	(226)		20.3 %	(320)					*P* < 0.0001
	CEPVR	Day 0	0.0 %	(136)	*P* > 0.05	0.0 %	(136)	*P* < 0.0001				*P* > 0.05
		Day 7	0.0 %	(226)		15.3 %	(320)					*P* < 0.0001
age-1(hx546)	CEPDL	Day 0	0.0 %	(130)	*P* > 0.05	0.0 %	(123)	*P* > 0.05	*P* > 0.05			*P* > 0.05
		Day 7	0.0 %	(136)		4.4 %	(297)		*P* > 0.05			*P* = 0.05
	CEPDR	Day 0	0.0 %	(130)	*P* > 0.05	0.0 %	(123)	*P* = 0.002	*P* > 0.05			*P* > 0.05
		Day 7	0.0 %	(136)		7.4 %	(297)		*P* > 0.05			*P* = 0.001
	CEPVL	Day 0	0.0 %	(130)	*P* > 0.05	0.0 %	(123)	*P* < 0.0001	*P* > 0.05			*P* > 0.05
		Day 7	0.0 %	(136)		33.3 %	(297)		*P* = 0.001			*P* < 0.0001
	CEPVR	Day 0	0.0 %	(130)	*P* > 0.05	0.0 %	(123)	*P* < 0.0001	*P* > 0.05			*P* > 0.05
		Day 7	0.0 %	(136)		31.6 %	(297)		*P* < 0.0001			*P* < 0.0001
daf-16(mu86); age-1(hx546)	CEPDL	Day 0				0.0 %	(93)	*P > 0.05*		*P* > 0.05		
		Day 7				4.9 %	(203)			*P* > 0.05		
	CEPDR	Day 0				1.0 %	(93)	*P > 0.05*		*P* > 0.05		
		Day 7				5.9 %	(203)			*P* > 0.05		
	CEPVL	Day 0				0.0 %	(93)	*P* = 0.002		*P* > 0.05		
		Day 7				10.3 %	(203)			*P* < 0.0001		
	CEPVR	Day 0				0.0 %	(93)	*P* = 0.002		*P* > 0.05		
		Day 7				10.3 %	(203)			*P* < 0.0001		
daf-16(mu86); age-1(hx546); Pgut::daf-16(+)	CEPDL	Day 0				1.0 %	(179)	*P* = 0.002			*P* > 0.05	
		Day 7				9.4 %	(233)				*P* > 0.05	
	CEPDR	Day 0				0.0 %	(179)	*P* < 0.0001			*P* > 0.05	
		Day 7				9.4 %	(233)				*P* > 0.05	
	CEPVL	Day 0				0.0 %	(179)	*P* < 0.0001			*P* > 0.05	
		Day 7				10.7 %	(233)				*P* > 0.05	
	CEPVR	Day 0				0.0 %	(179)	*P* = 0.0001			*P* > 0.05	
		Day 7				7.7 %	(233)				*P* > 0.05	

Table S5: Percent of cephalic neurons missing in wild type and *daf-2(e1370)* mutants expressing α-synuclein in dopaminergic neurons.

			Day 0			Day 7			
α-synuclein	Genotype	Cell	Cells missing (%)	n	Comparisons to wild type (Fisher's Exact test)	Cells missing (%)	n	Comparisons to wild type (Fisher's Exact test)	Comparisons across age (Fisher's Exact test)
Yes	wild type	CEPDL	0.0 %	(114)		16.0 %	(213)		*P* < 0.0001
		CEPDR	0.0 %	(114)		16.0 %	(213)		*P* < 0.0001
		CEPVL	0.0 %	(114)		6.6 %	(213)		*P* = 0.006
		CEPVR	0.0 %	(114)		6.6 %	(213)		*P* = 0.01
	daf-2(e1370)	CEPDL	0.0 %	(106)	*P* > 0.05	4.3 %	(304)	*P* < 0.0001	*P* > 0.05
		CEPDR	0.0 %	(106)	*P* > 0.05	5.9 %	(304)	*P* = 0.0006	*P* = 0.01
		CEPVL	0.9 %	(106)	*P* > 0.05	18.1 %	(304)	*P* = 0.0002	*P* < 0.0001
		CEPVR	1.9 %	(106)	*P* > 0.05	15.5 %	(304)	*P* = 0.004	*P* < 0.0001

Experiment conducted at 20ºC. Animals cultured in 20 μM FUDR starting at L4 molt. *fer-15(b26)* not present in the genetic background.
